# Interpretation of *Euphorbia Kansui* Stir-Fried with Vinegar Treating Malignant Ascites by a UPLC-Q-TOF/MS Based Rat Serum and Urine Metabolomics Strategy Coupled with Network Pharmacology

**DOI:** 10.3390/molecules23123246

**Published:** 2018-12-07

**Authors:** Yi Zhang, Jing Gao, Qiao Zhang, Kan Wang, Weifeng Yao, Beihua Bao, Li Zhang, Yuping Tang

**Affiliations:** 1 Jiangsu Key Laboratory for High Technology Research of TCM Formulae, National and Local Collaborative Engineering Center of Chinese Medicinal Resources Industrialization and Formulae Innovative Medicine and Jiangsu Collaborative Innovation Center of Chinese Medicinal Resources Industrialization, Nanjing University of Chinese Medicine, Nanjing 210023, China; zhangyi@njucm.edu.cn (Y.Z.); gaojing@njucm.edu.cn (J.G.); 18700081184@163.com (Q.Z.); wangkan86@hotmail.com (K.W.); scotter01@163.com (B.B.); 2 Key Laboratory of Shaanxi Administration of Traditional Chinese Medicine for TCM Compatibility, Shaanxi University of Chinese Medicine, Xi’an 712046, China; yupingtang@sntcm.edu.cn

**Keywords:** Kansui stir-fried with vinegar, metabolomics, malignant ascites, UPLC-QTOF/MS, network pharmacology

## Abstract

*Euphorbia kansui* stir-fried with vinegar (V-kansui) has promising biological activities toward treating malignant ascites with reduced toxicity compared to crude kansui. But the mechanism concerning promoting the excretion of ascites has not been systematically studied. The purpose of this paper was to investigate the possible mechanism of V-kansui in treating malignant ascites, including metabolic pathways and molecular mechanism using an integrated serum and urine metabolomics coupled with network pharmacology. Serum and urine samples of rats were collected and analyzed by ultra-high-performance liquid chromatography coupled with quadrupole time-of-flight mass spectrometry (UPLC-Q-TOF/MS). A comparison with crude kansui was also made to demonstrate the feasibility of processing. Principle component analysis (PCA) and orthogonal partial least square discriminate analysis (OPLS-DA) were conducted to discriminate the groups, search important variables and reveal the possible pathways. A compound-target-metabolite network was finally constructed to identify the crucial targets to further understand the molecular mechanism. Sixteen significant metabolites contributing to the discrimination of model and control groups were tentatively screened out. They were mainly involved in the arachidonic acid metabolism, steroid hormone biosynthesis and primary bile acid to possibly reduce inflammatory and modulate the renin-angiotensin-aldosterone system to achieve treating malignant ascites. A bio-network starting from the compounds and ending in the metabolites was constructed to elucidate the molecular mechanism. HSP90AA1, ANXA2, PRDX6, PCNA, SOD2 and ALB were identified as the potential key targets that were responsible for the treatment of malignant ascites by the parameter combining the average shortest path length and betweenness centrality. The correlated 17 compounds were considered as the potential active ingredients in V-kansui. In addition, the metabolomics showed that the effect of V-kansui was almost in accordance with crude kansui. These results systematically revealed the mechanism of V-kansui against malignant ascites for the first time using metabolomics coupled with network pharmacology. V-kansui could be a promising safe and therapeutic medicine for the excretion of ascites.

## 1. Introduction

Hepatocellular carcinoma (HCC) is attracting much more attention because it can lead to most malignancies. In recent years, the incidence of HCC dramatically increases [1]. Although advances have been made in the treatment of HCC, the prognosis of which remains unsatisfactory. Malignant ascites is one of the severe complications of HCC. Besides, malignant ascites is the end stage of malignancy and greatly affects the deterioration of the patient’s life [2]. Current treatments mostly focus on the elimination of ascites, such as salt restriction and peritoneal catheter drainage. Salt restriction has a high risk of protein malnutrition due to the reduced nutrient intake, which may result in sarcopenia and increased mortality. Besides, large volume paracentesis is invasive, requires more time of recovery and leads to bad quality of life. Diuretic therapy after large volume paracentesis is also not sufficient to prevent the recurrence of ascites. And the most frequent side effects of diuretics are hyponatremia, intravascular volume depletion and hepatic encephalopathy [3]. Hence, the results of the above methods are still unfavorable and the treatment of malignant ascites is an urgent problem that need to be solved.

Kansui, the dried root of *Euphorbia kansui* S.L. Liou ex S.B. Ho, is a commonly used Chinese herbal medicine (CHM) and is recorded in *Sheng Nungs Herbal* for multiple medical applications [4]. As we know, kansui has promising biological effects on cancer [5], pancreatitis [6], immune regulation [7], intestinal obstruction [8] and diabetes [9]. Among these, kansui is particularly applied to edema, ascites and asthma [10]. But the severe toxicity to liver and kidney greatly restricts its clinical practice [11]. Stir-fried with vinegar (V-kansui) was confirmed to reduce the toxicity without compromising the efficacy of kansui on the basis of ancient people’s experiences and advances in modern technologies [12]. Our previous study has compared the effect of expelling water retention with crude kansui and V-kansui on cancerous ascites rats. The volumes of pleural fluid significantly decreased in the V-kansui group by increasing the volumes of urine in model rats. Serum biochemical study also showed that the levels of PRA, Ang II, ALD and ADH declined (*p* < 0.05), indicating the remarkable effect of V-kansui on the treatment of malignant pleural effusion [13]. Large numbers of efforts have been made to investigate how crude kansui and V-kansui treat the malignant ascites. To date, nearly 100 compounds have been extracted and identified from kansui [14]. Among these, diterpenes and triterpenes were recognized as the main chemical constituents because rats given these two fractions showed a significant increase in urine and a decrease in ascites [15]. A sort of diterpene, kansuinine B, can inhibit the IL-6 induced signal transduction by activating the ERK 1/2 and lead to increasing the expression of STAT3 serine phosphorylation and SOCS-3 [16]. And euphol, a triterpene in kansui, induced the ERK 1/2 activation to promote the apoptosis of human gastric cancer cells [17]. Recent study also demonstrated that ingenane-type diterpene compounds from kansui induced IFN-γ secretion and activated the translocation of p65 to the nucleus in natural killer cells [5]. CHMs are composed of multiple components with diverse structures that are against multiple targets [18]. To our best of knowledge, the systematic mechanism containing active compounds, targets and metabolites of malignant ascites with V-kansui treatment remains unclear.

The ‘-omics’ techniques have been popular methods to explore the mechanism and evaluate the safety of CHMs in recent years [19]. Metabolomics is a branch of system biology with a knowledge of multiple subjects, such as biology, chemistry and informatics. It describes the collective characterizations in biological, physiological and pathological conditions after ectogenic stimulation [20]. It systematically identifies the endogenous small molecule metabolites and quantitatively analyzes the concentration changes in response to the disturbances, which represents the “wholeness,” “dynamic concept” and “dialectics” of CHM features [21]. Nowadays, with the development of modern analytical technologies, nuclear magnetic resonance (NMR), gas chromatography-mass spectrometry (GC-MS) and liquid chromatography-mass spectrometry (LC-MS) are employed in the metabolomics combined with multivariate analysis, such as PCA, PLS-DA and OPLS-DA [22,23,24]. Among these methods, ultra-high performance liquid chromatography with quadrupole-time-of-flight (UPLC-Q-TOF) has high resolution, selectivity and accuracy, making it possible to be applied to the complex, particularly for CHMs [25]. Blood and urine samples are prone to be collected and become the most attractive biofluids in metabolomics studies [26]. Metabolite profiles of serum can be regarded as important indicators of physiological and pathological states and may aid in the understanding of the mechanism behind disease occurrence and progression on the metabolic level. Urine can also contain disease biomarkers, specifically, excreted metabolites. Serum samples usually represent for low polar metabolites, while urine samples are composed of high polar ones. These two complement each other and stand for the globe features to obtain comprehensive understandings about the mechanism of drug, prognosis and sensitivity prediction to clinical treatments [27,28,29]. However, a myriad of metabolites and their broad range of concentrations limit the understanding of biological process of disease using the metabolomics alone. The complicated action mechanism always contains a plenty of biochemical reactions and signal paths from the original CHM to the final metabolites [30]. Network pharmacology combining systematic network with pharmacology could relate the endogenous metabolites to the targets, providing insights into the mechanism of CHMs that are a class of multiple-compounds and multiple-targets substances to reveal the drug-disease mechanism and molecular understandings systematically [31,32,33]. 

Therefore, in this study, an integrated UPLC-Q-TOF/MS based serum and urine metabolomics strategy coupled with network pharmacology was established to illustrate how V-kansui modulated the malignant ascites. The potential endogenous metabolites were screened by multivariate data analysis and the corresponding metabolic pathways were further determined to explore the functions of V-kansui. Then, the compound-target-metabolite network was constructed to identify the key targets of malignant ascites by the crucial compounds in V-kansui. In addition, a comparison with kansui was also made to intuitively demonstrate the feasibility of processing.

## 2. Results and Discussion

### 2.1. Metabolic Profiling of UPLC-Q-TOF/MS

Metabolic profiling of serum and urine samples were obtained in both positive and negative ion modes. The representative based peak intensity (BPI) chromatograms of serum and urine are displayed in Figure S1 and Figure S2. 377 and 504 metabolites were detected from serum and urine samples, respectively. They were separated well within 15 min. Besides, obvious changes with respect to the contents of some metabolites could be observed in both ion modes. Then, the complexity of the chromatography and individual differences among the groups were further analyzed using multivariate data analysis, such as PCA and OPLS-DA.

### 2.2. Multivariate Data Analysis

**Figure 1:**
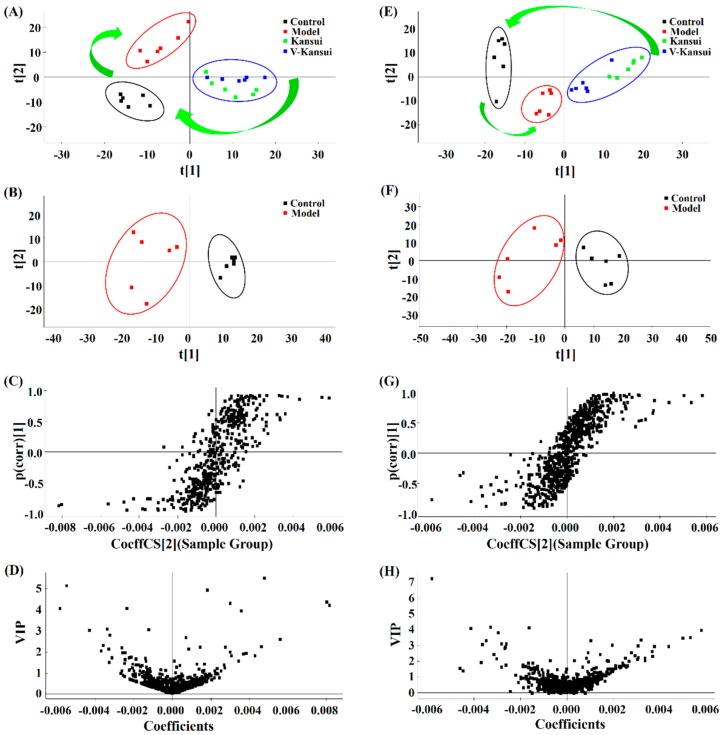
The PCA score plots (**A**,**E**) of four groups, PCA score plots (**B**,**F**), S-plots (**C**,**G**) and VIP-plots (**D**,**H**) of OPLS-DA between control and model groups in positive ESI mode of serum and urine samples. **A**–**D** represents the serum samples while **E**–**H** represents the urine samples.

After the Pareto normalization of the data from the serum and urine samples of four groups, PCA was firstly employed using SIMCA-P 12.0 demo software. The numbers of the principle components were auto fit to meet the specification of PCA analysis. The PCA models built by 377 serum metabolites and 504 urine metabolites for positive modes captured 65.0% and 68.6% cumulative variance of the independent variable (R^2^_X_) with 4 and 5 principle components, separately. These demonstrated that the models had good explanatory ability. The score plots showed how the samples distributed in the latent variable space and were employed to elucidate the tendency of the four groups. From Figure 1A,E, the control group and model group spread apart. V-kansui group located between the model and control groups and was in close proximity to the kansui group. This indicated that V-kansui was adjusting the abnormal metabolic rats to the normal state, the efficacy of which was almost the same as crude kansui.

PCA score plots of control group and model group exhibited good separation of these two groups (Figure 1B,F), indicating the obvious differences in their metabolic distributions. Supervised OPLS-DA models were then built for both positive and negative modes of serum and urine samples. The models built by serum data for positive and negative modes could explain the 97.75% and 97.83% variance of the response variable (R^2^_Y_) and the cumulative explained variance for modeling in cross-validations (Q^2^) were 82.81% and 87.42%, separately. For urine data in positive and negative modes, R^2^_Y_ were 97.75% and 97.83% with Q^2^ 79.56% and 77.73%, individually. These demonstrated that the models had good explanatory and predictive ability. The permutation tests (*n* = 200) were employed to validate the predictive ability of the built OPLS-DA models. Results showed that all the R^2^ and Q^2^ values in OPLS-DA models were lower than in permutation tests (Figure S3). This demonstrated the goodness of fit and better predictive capability for the OPLS-DA models. The S-plot was then utilized to investigate the inherent clustering variables. Apparent variances between the endogenous metabolites from the control group and model group could be seen from Figure 1C,G. And the variables that located on the left down corner and the right top corner made significant contributions to the separation of the two groups. The variable importance in the projection (VIP) plots were further employed to determine the potential markers. Variables whose VIP values are over 1 means that they are the potential classification factors. In Figure 1D,H, differences between the two groups of control and model groups were consistent with that in the S-plot.

### 2.3. Potential Metabolites Identification

In order to make more precise identification, potential metabolites were selected based on the principles that VIP > 1.5 and *p*-value < 0.05 in the S-plot extracted between the control group and model group. All the candidate markers with exact *m*/*z* values were screened from the S-plot and VIP plot at this threshold. The accurate mass and fragments of the metabolite candidates were matched with the online database including HMDB (www.hmdb.ca), METIN (metlin.scipps.edu) and KEGG (www.kejj.jp). The mass tolerance between the measured *m*/*z* values and the exact mass was defined within 10 ppm. Finally, combined with online databases and literatures, the metabolites were identified.

**Figure 2:**
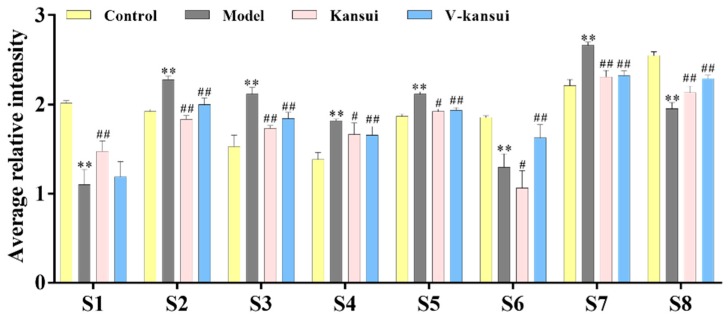
The average relative intensity changes of serum endogenous metabolites from different groups. Comparing the model group with the control group, ** indicates *p* < 0.01.; Comparing the kansui group and V-kansui group with the model group, ^#^ indicates *p* < 0.05, and ^##^ indicates *p* < 0.01 indicates *p* < 0.05.

**Figure 3:**
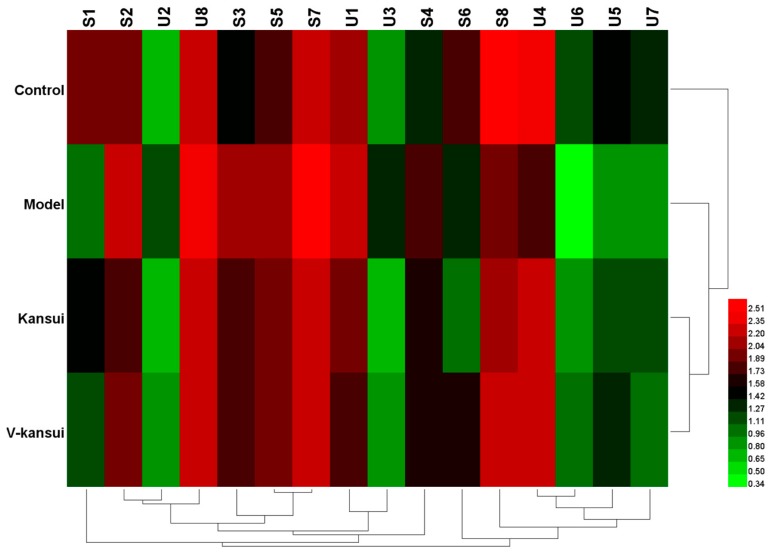
The heatmap of 16 potential metabolites.

For serum, eight endogenous metabolites were screened out (detailed information see Table 1). Compared to the control group, the levels of taurocholic acid (TCA), taurochenodesoxycholic acid (TCDA), cholic acid (CA), phytosphingosine and LysoPC significantly increased, while the levels of indoleacetaldehyde, chenodeoxycholic acid (CDCA) and docosahexaenoic acid (DHA) significantly decreased in the model group (*p* < 0.05). It indicated that metabolic disorders occurred in the normal rats after the injury. The average relative intensities of these metabolites were displayed in Figure 2 to intuitively visualize the effect of V-kansui on them. The average relative intensity of metabolites in the V-kansui group had significant differences over those in the model group (*p* < 0.05), showing a great degree of recovery. Compared with the kansui group, the adjustment of metabolic disorders by V-kansui group was nearly the same degree and they both were close to the control group. For urine samples, eight endogenous metabolites were identified according the protocol details above (see Table 1). In comparison of the model group to the control group, Prostaglandin G_2_ (PGG_2_), 10-Formyl-THF and phytosphingosine significantly increased and 5-Hydroxy-6-methoxyindole glucuronide/6-Hydroxy-5-methoxyindole glucuronide, 5-*l*-glutamyl-taurine, riboflavin, androstenedione and 11beta-hydroxyprogesterone significantly decreased (*p* < 0.05). The average relative intensity of these metabolites appeared to be the same phenomenon as seen in Figure S4 that the treatment of kansui stir-fried with vinegar was equivalent to kansui when improving the safety in clinic. The heatmap (Figure 3) displayed the distribution patterns of 16 potential metabolites among the four groups using Heml software. Vertical cluster analysis demonstrated the differences between the model and control groups and the equivalent efficacy of V-kansui to kansui.

### 2.4. Metabolic Pathway Analysis

**Figure 4:**
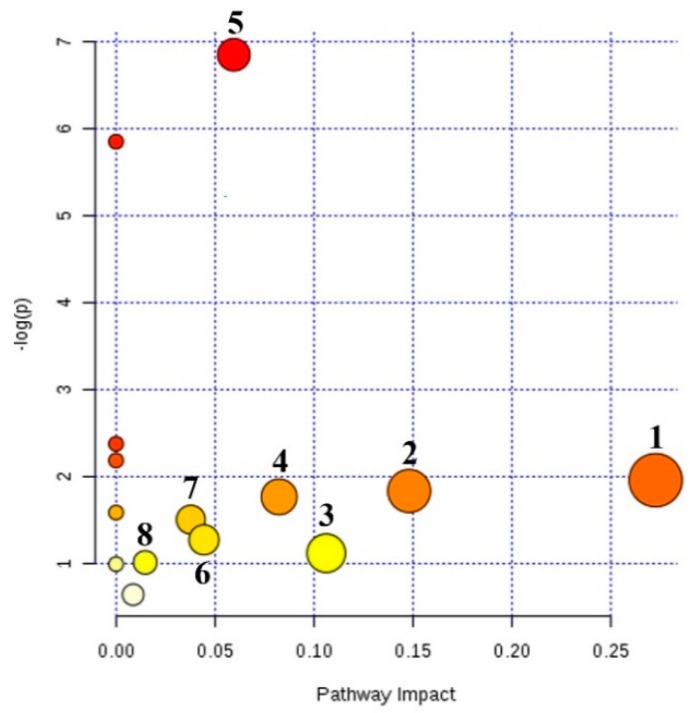
Summary of metabolic pathway analysis of potential metabolites. 1. Pentose and glucuronate interconversions; 2. Glyoxylate and dicarboxylate metabolism; 3. Arachidonic acid metabolism; 4. Steroid hormone biosynthesis; 5. Primary bile acid biosynthesis; 6. Glycerophospholipid metabolism; 7. Starch and sucrose metabolism; 8. Tryptophan metabolism.

In order to explore the mechanism of V-kansui on the malignant ascites, the metabolic pathways were constructed by importing the identified potential metabolites into MetaboAnalyst and fourteen pathways were obtained (Figure 4). Among these, eight pathways, that is, pentose and glucuronate interconversions, glyoxylate and dicarboxylate metabolism, arachidonic acid metabolism, steroid hormone biosynthesis, primary bile acid biosynthesis, glycerophospholipid metabolism, starch and sucrose metabolism and tryptophan metabolism, played key roles in reflecting the changes of serum and urine metabolites with the impact-value 0.27, 0.15, 0.11, 0.08, 0.06, 0.04, 0.03 and 0.01, respectively.

### 2.5. Network Pharmacology

To gain more insights into the mechanism, the KEGG IDs, fold change and *p*-value of potential metabolites were loaded into the MetScape to construct the gene-metabolite network (Figure S5). These 113 proteins were extended to adjacent proteins using the BioGenet to further explore the relationship between the metabolite-related proteins and disease targets. From Figure S6, the protein-protein network included 825 adjacent proteins that may have potential correlations to the disease targets.

A total of 31 compounds were identified in the V-kansui (see Table 2). Then they were screened by absorption, distribution, metabolism and excretion (ADME) model with the values of oral bioavailability (OB) and drug-likeness (DL). 8 of these met with the criteria that had OB ≥ 30% and DL ≥ 0.18 [34]. In addition, 13 compounds below this specification (GE05-GE08, GE12, GE14, GE15, GE17, GE18 and GE24-GE27) were also taken as the active compounds that had promising treatment to the malignant ascites [4,5,16,35]. Thus, a total of 21 compounds were selected for further analysis.

There were 566 genes significant to the HCC integrating the OncoDB.HCC [36] and Liversome databases [37]. The targets of the 21 active compounds were collected from PharmMapper (http://lilab.ecust.edu.cn/pharmmapper/index.php) and ChemMapper (http://lilab.ecust.edu.cn/chemmapper/). Then the compound-target network for the effect of treating malignant ascites by V-kansui was built using the union 80 proteins and shown in Figure S7.

**Figure 5:**
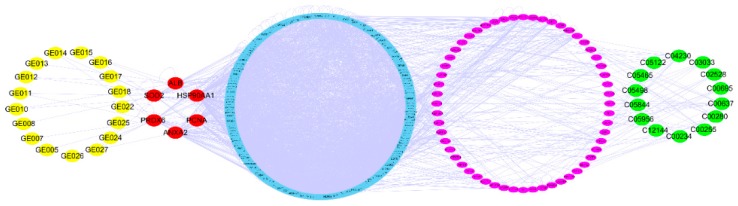
The crucial compound-target-metabolite network. The yellow, red, blue, pink and green nodes represent the active compounds, targets, adjacent proteins, pathway genes and potential metabolites, respectively.

The compound-target-metabolite network was built integrating the above three network (Figure S8). This indicated the whole biochemical process from the V-kansui to the metabolites. The R value was calculated by the parameters containing average shortest path length and betweenness centrality to determine the key targets. Targets with a low average shortest path length and high betweenness centrality played important roles in the network. The R values of 12 intersection proteins from the compound-target and protein-protein-metabolites were shown in Table 3. HSP90AA1, ANXA2, PRDX6, PCNA, SOD2 and ALB were crucial disease targets (Figure 5). The correlated 17 compounds were considered as the potential active ingredients in V-kansui.

### 2.6. Discussion

In this study, a UPLC-Q-TOF/MS based rat serum and urine metabolomics was firstly employed to interpret the mechanism of V-kansui treating malignant ascites. A total of 16 differential metabolites were confirmed and involved in 8 main metabolic pathways. The glucuronide pathway is an important phase II metabolic elimination reaction, which is vital for the clearance of the oxidative metabolites, toxins, endogenous hormones and cholic acid [38,39]. V-kansui promoted the excretion of the oxidative metabolites and toxic substances to alleviate the liver injuries [40] through the urine, which appeared to be a significant increase of the level of 5-hydroxy-6-methoxyguanidine compared to the model rats.

Arachidonic acid is catalyzed by cyclooxygenase (COX) to generate prostaglandins (PGs), which regulate fever, inflammation, smooth muscle contraction and translocation of water and salt in the kidney [41,42]. PGG_2_ is then converted into PGE_2_ under the catalysis of PGE_2_ synthase. PGE_2_ modulates renal blood flow and glomerular filtration rate, affect the water and sodium transport of distal renal tubules and stimulate renin to release from the bypass of glomerulus [43]. The renin acts on the renin-angiotensin-aldosterone system to convert angiotensinogen to angiotensin and adrenal cortex is strongly stimulated to secrete aldosterone to improve the reabsorption of water and sodium [44]. Liver damage and inflammatory reactions also relate to the synthesis of steroid hormones [45]. The pregnenolone is typically generated by the cholesterol under the Cytochrome P450 (CYP450) and converted to 11beta-hydroxy progesterone. 11beta-hydroxy progesterone could hamper the enzyme 11beta-hydroxysteroid dehydrogenase that is the inhibitor in the regulation of glucocorticoid-induced Na^+^ retention [46]. Compared to the control group, the high level of PGG_2_ and the low levels of 11beta-hydroxy progesterone and androstenedione in model rats exhibited inflammatory reactions and abnormal water-electrolyte metabolism, leading to ascites tumors. V-kansui mainly treated these symptoms through a bidirectional regulatory mechanism. On one hand, V-kansui decreased the level of PGG_2_ to enhance the permeability of the peritoneal capillary and inhibit the renin-angiotensin-aldosterone system to excrete the over excess production of water and electrolyte. On the other hand, the metabolism of adrenocortical hormone was corrected by increasing the level of the 11beta-hydroxy progesterone and androstenedione, enhancing the metabolism of water.

The changes in the levels of phospholipids can also be taken as indicators for liver injury [47]. Phospholipids can be divided into phosphoglyceride (PC) and sphingomyelin. Phytosphingosine is one of the biomarkers for liver cancer and the accumulation of sphingosine is related to the apoptosis of hepatocytes [48,49,50]. PC is hydrolyzed under the catalysis of phospholipase A2 and then fatty acids in the position sn-2 are released, finally transforming into Lysophosphoglyceride (LPC) [51]. LPC has cytotoxicity and it can increase the permeability of vascular endothelial cells and promote the inflammatory response, leading to structural and functional damage to the vascular endothelium [52,53]. Bile acids are also critical to the digestion and absorption of fat [54]. Damaged liver cells can cause high activity of cholesterol 7-hydroxylase and 12-hydroxylase in hepatocytes, decreasing the removal of bile acids. A recent study demonstrated that the commensal gut bacterial could balance the metabolism of primary bile acids to stimulate the expression of CXCL16 to promote the accumulation of natural killer T (NKT) cells in the liver, finally prohibiting the growth of the malignant tumor [55]. In the rats administered by intraperitoneal injection with Walker 256 cells, the content of LPC and phytosphingosine notably increased, suggesting the inflammatory injuries. A remarkable decrease of LPC and phytosphingosine was observed in the serum and urine of the V-kansui group and the levels were similar to the control group. And the level of CDCA, which positively correlated with the CXCL16 expression was increased by V-kansui, demonstrating protective effect on the injured liver.

Tryptophan is an essential human amino acid and regulates the synthesis of proteins [56]. NADPH is released to provide energy for the growth of tumor [57], when methylenetetrahydrofolate is oxidized to 10-formyl-tetrahydrofolate (10-formyl-THF) by co-enzyme tetrahydrofolate. High content of 10-Formyl-THF in the urine of the model rats were significantly measured, showing the abnormal energy metabolism and DNA synthesis [58]. V-kansui enhanced the indole acetaldehyde levels in rat serum and restrained the generation of NADPH and electron transfer, declining the energy supply.

In order to understand the mechanism comprehensively, a bio-network starting from the compounds in V-kansui and ending in metabolites was established. The crucial targets of V-kansui treating the malignant ascites were identified by the parameter integrating the average shortest path length and betweenness centrality, that is, HSP90AA1, ANXA2, PRDX6, PCNA, SOD2 and ALB. HSP90AA1 is now an applied biomarker of detecting HCC. HSP90 enhanced the glycolysis and proliferation and inhibited the apoptosis of HCC cells through PKM_2_ [59]. ANXA2 is a calcium-dependent phospholipids binding protein, which expressed highly in HCC cells. ANXA2 could promote the malignant behavior by remodeling the motility structures [60]. PRDX6 and SOD2 were identified as antibodies in patients with HCC by a new type of protein chip [61]. In primary HCC, PCNA was not only involved in the proliferation but also participated in the DNA repair with P21, making it a promising potential diagnostic biomarker [62]. High level of ALB in serum was the indicator of satisfactory prognosis. Nojiri et al. showed that ALB could inhibit the HCC proliferation and enhance the number of G0/G1 cells [63]. The correlated 17 compounds were considered as the potential active ingredients. Our previous studies have demonstrated the efficacy of different section of V-kansui to malignant ascites. The active cite was in the part of ethyl acetate. In it, ingenane-type and jastrophane-type diterpenoids played important roles in the treatment of malignant ascites [15]. Therein, 11 compounds are ingenane-type and jastrophane-type diterpenoids and triterpenoids. Modern pharmacological study showed that kansuiphorin B and kansuinin A that belong to the ingenane-type and jastrophane-type diterpenoids, respectively, could inhibit NF-kB activity to exert anti-inflammatory activity in RAW264.7 macrophage cells based on a bioassay-guided separation [64].

## 3. Materials and Methods

### 3.1. Materials, Chemicals and Reagents

Acetonitrile of HPLC grade and analytical formic acid were purchased from Merck (Darmstadt, Germany). Ultrapure water was filtered by a Milli-Q super purification system (Milford, MA, USA). Other reagents and chemicals were of analytical grade.

The roots of *Euphorbia kansui* T.N. Liou ex T.P. Wang were purchased from Baoji, Shanxi Province, China and were identified by Professor Qinan Wu (School of Pharmacy, Nanjing University of Chinese Medicine, Nanjing, China). The voucher specimens (NJUCM-20151020) were stored in the Herbarium of Nanjing University of Chinese Medicine.

### 3.2. Preparation of Samples of CHM Pieces

Crude kansui was adequately soaking with 30% vinegar until the solvent could not be absorbed by it. Then the vinegar-processed kansui was acquired when slight scorched spots appeared on the surface through placing it in an environment at 260 °C (Chinese Pharmacopeia, 2015 edition). Both V-kansui and crude kansui were milled into powders (65-mesh) before use.

### 3.3. Animals and Treatment

Male SD rats (*n* = 24) were bought from the Shanghai Xi Purr-will Kay Experimental Animal Co., Ltd. All rats were kept in the controlled environmental conditions: 12 h light/12 h dark cycle; temperature, 25 °C; relative humidity, 30–45%. They were given a standard diet and water ad libitum. All rats were fed to acclimatize for a week and then were randomly divided into four groups (*n* = 6/group) as follows: Control, Model, Kansui and V-Kansui. Except for Control, rats in remaining groups were injected intraperitoneally with Walker 256 cells (1 × 10^7^/mL, 1 mL per rat). From the next day, Control and Model groups were orally administrated with 0.5% CMC-Na and rats in Kansui and V-kansui groups were orally administrated with raw kansui and V-kansui at a dose of 1 mL per 100 g. The concentrations of kansui and V-kansui were both 680 mg·kg^−1^ that corresponded to 8 times the clinical dosage [65]. All groups were given intra gastric administrations once a day for a week. Animal care was in accordance with the Guidelines for Animal Experimentation of Nanjing University of Chinese Medicine and protocols approved by the Animal Ethics Committee of the institution.

### 3.4. Collection and Preparation of Serum and Urine Samples

Blood samples were collected from the common carotid artery of rats in the 7th day after administration. Serums were then acquired by segregating the supernatant from the blood after setting aside for thirty minutes at room temperature and centrifuging at 4000 rpm for 10 min (5811 Eppdendorf refrigerated centrifuge, Germany). Then they were stored at −80 °C before use.

Urine samples were collected after twelve hours at the seventh day of oral administration, lasting for 12 h. Samples were centrifuged at 4000 rpm for 10 min. Then the supernatants were stored at −80 °C before use.

All frozen serum samples were thawed and equilibrated at 4 °C before conducting analysis. Afterwards, 800 μL acetonitrile was added into 200 μL aliquots of serum samples. The mixture was vortex-mixed for 2 min and centrifuged at 13,000 rpm for 10 min (5811 Eppdendorf refrigerated centrifuge, Germany). Then, 850 μL supernatants of serum samples were evaporated to dryness in a vacuum by centrifugation at 30 °C. The dried residues were dissolved with 200 μL of acetonitrile and water (70:30, *v*/*v*), vortex-mixed for two minutes and centrifuged at 13,000 rpm for 10 min. Finally, 2 μL aliquots of supernatant were injected to UPLC–TOF/MS for analysis. The procedures of urine samples were of the same.

### 3.5. Chromatography Conditions

Chromatographic analysis of serum and urine samples was performed on an ACQUITY UPLC C18 column (100 mm×2.1 mm, 1.7 μm; Waters, USA) using a UPLC Acquity^TM^ system (Waters, Williamsburg, VA, USA). The column temperature was 35 °C and the flow rate was 0.4 mL·min^−1^. The injection volume was 2 μL. The mobile phase was consisted of 0.1% formic acid in water (A) and acetonitrile (B) by gradient elution. The gradient elution of serum samples were as follows: 0–3 min, 5%–45% B; 3–13.5 min, 45%–95% B; 13.5–14.5 min, 95% B; 14.5–15.0 min, 95–5% B. While the gradient elution conditions of urine samples were 0–8min, 5–30% B; 8–11 min, 30–70% B; 11–13 min, 70–95% B; 13–14 min, 95% B; 14–15 min, 95–5% B. 20 μL of each serum and urine sample were mixed to obtain a quality control (QC) sample, respectively. The QC sample was injected every ten samples to monitor the consistency of the system.

### 3.6. Mass Spectrometry

Mass spectrometry of both serum and urine samples was conducted on a Waters Synapt High Definition TOF Mass system (Waters Corp., Milford, MA, USA) accompanied with electrospray ionization source. The ESI source in both positive and negative ion mode were selected to monitor for analysis. The capillary voltage was 3.0 kV and the sampling cone voltage was 30.0 V. The extraction cone voltage was kept at 1.0 V. The source temperature was set at 120 °C and desolvation gas temperature was 350 °C. The flow rates of cone and desolvation gas were 50 L·h^−1^ and 600 L·h^−1^. Nitrogen and argon were taken as cone and desolvation gas, individually. The rate of data acquisition was set to 0.5 s with a 0.02 s inter scan delay under the collision energy of 6 eV. The scanning mass range was from 100 to 1000 Da. For accurate mass acquisition, leucine enkephalin at a concentration of 0.2 pg·mL^−1^ was used as a lock mass solution at a flow rate of 100 μL·min^−1^, monitoring for positive ion mode ([M + H]^+^ 556.2771) and for negative ion mode ([M − H]^−^ 555.2615).

### 3.7. Data processing and Pathway Analysis

The raw mass data were analyzed with MassLynx v4.1 and MarkerLynx Application Manager (Waters Corp., Milford, MA, USA) for peak extraction, alignment and normalization. Multivariate analysis was realized by introducing the resultant data to EZinfo software 2.0, that is, principle component analysis (PCA) and orthogonal projection to latent structures-discriminant analysis (OPLS-DA). Pathway analysis was performed on MetaboAanalyst 4.0, a web tool combined with the KEGG.

### 3.8. Network Analysis

In order to make comprehensive understandings of the mechanism concerning V-kansui treating malignant ascites, the compound-target-metabolite network was established. The potential integrated serum and urine metabolites and corresponding genes were visualized by the MetScape plugin. Then the protein-protein interactions were displayed by importing these related genes into the BioGenet. The HCC-related genes were obtained combining the two liver databases OncoDB.HCC and Liverome. Chemical compounds in V-kansui were searched and confirmed by TCMSP database (http://lsp.nwu.edu.cn/tcmsp.php). Targets of these selected compounds were obtained combining ChemMapper and PharmMapper based on 3D similarity. Finally, a bio-network compound-disease-gene-metabolite was then constructed by Cytoscape software 3.6.1. Average shortest path length and betweenness centrality auto-calculated by NetworkAnalyzer were to determine the R value to rank the targets by the equation as follows [66]:

(1) $$R\;=\;\frac{A_{i}-A_{i}\left(min\right)}{A_{i}\left(max\right)-A_{i}\left(min\right)}\times 50\%+\frac{\frac{1}{B_{j}}-\frac{1}{B_{j}}\left(min\right)}{\frac{1}{B_{j}}\left(max\right)-\frac{1}{B_{j}}\left(min\right)}\times 50\%$$

where A_i_ is the average shortest path length and B_j_ is the betweenness centrality.

## 4. Conclusions

In this study, integrated serum and urine metabolomics based on UPLC-Q-TOF-MS coupled with network analysis was used to interpret the mechanism of kansui stir-fried with vinegar in treating malignant ascites. A total of sixteen potential metabolites were identified to be mainly involved in the arachidonic acid metabolism, steroid hormone biosynthesis and primary bile acid metabolism to possibly reduce inflammatory and modulate the renin-angiotensin-aldosterone system to achieve treating malignant ascites. Twenty compounds were employed for the network analysis and the key targets responsible for the treatment of V-kansui were HSP90AA1, ANXA2, PRDX6, PCNA, SOD2 and ALB. In addition, combined with previous biochemical analysis, the metabolomics showed that the effect of V-kansui was almost in accordance with crude kansui with improved clinical safety. This study also demonstrated that metabolomics coupled with network analysis is a powerful approach to investigate the mechanism of V-kansui against malignant ascites.

## Figures and Tables

**Table 1:** Identification results of potential serum and urine metabolites by UPLC-Q-TOF-MS.

No.	RT	*m*/*z*	Mass Error (ppm)	VIP	Metabolites	Adduct	Formula	KEGG	Trend	Pathway
S1	1.18	160.0698	8.7	2.6	Indoleacetaldehyde	M + H	C_10_H_9_NO	C00637	↓	Tryptophan metabolism
S2	2.88	514.2942	5.0	1.6	Taurocholic acid	M − H	C_26_H_45_NO_7_S	C05122	↑	Primary bile acid biosynthesis, Taurine and hypotaurine metabolism
S3	3.91	498.2991	4.8	2.1	Taurochenodesoxycholic acid	M − H	C_26_H_45_NO_6_S	C05465	↑	Primary bile acid biosynthesis
S4	4.46	407.2886	2.4	5.2	Cholic acid	M − H	C_24_H_40_O_5_	C00695	↑	Primary bile acid biosynthesis
S5	4.79	318.2912	−5.3	1.9	Phytosphingosine	M + H	C_18_H_39_NO_3_	C12144	↑	Sphingolipid metabolism
S6	6.15	391.2934	2.0	2.1	Chenodeoxycholic acid	M − H	C_24_H_40_O_4_	C02528	↓	Primary bile acid biosynthesis
S7	8.27	522.3438	−8.2	2.3	LysoPC(18:1) ^a^	M + H	C_26_H_52_NO_7_P	C04230	↑	Glycerophospholipid metabolism
S8	10.88	327.2400	−0.6	2.8	Docosahexaenoic acid	M − H	C_22_H_32_O_2_	C06429	↓	Biosynthesis of unsaturated fatty acids
U1	3.73	340.0978	7.1	2.8	5-Hydroxy-6-methoxyindole glucuronide/6-Hydroxy-5-methoxyindole glucuronide	M + H	C_15_H_17_NO_8_	C03033	↓	Pentose and glucuronate interconversions
U2	6.46	413.2165	−9.0	1.5	Prostaglandin G_2_	M + FA − H	C_20_H_32_O_6_	C05956	↑	Arachidonic acid metabolism
U3	6.71	472.1692	7.0	1.8	10-Formyltetrahydrofolate	M − H	C_20_H_23_N_7_O_7_	C00234	↑	One carbon pool by folate, Glyoxylate and dicarboxylate metabolism, Aminoacyl-tRNA biosynthesis
U4	7.22	255.0563	−3.5	4.0	5-*L*-Glutamyl-taurine	M + H	C_7_H_14_N_2_O_6_S	C05844	↓	Taurine and hypotaurine metabolism
U5	8.96	377.1403	5.6	2.1	Riboflavin	M + H	C_17_H_20_N_4_O_6_	C00255	↓	Riboflavin metabolism
U6	9.07	331.1939	2.1	1.9	Androstenedione	M+FA-H	C_19_H_26_O_2_	C00280	↓	Steroid hormone biosynthesis
U7	9.64	331.1973	−4.2	1.7	11b-Hydroxyprogesterone	M − H	C_20_H_28_O_4_	C05498	↓	Steroid hormone biosynthesis
U8	10.48	318.2924	−1.6	2.6	Phytosphingosine	M + H	C_18_H_39_NO_3_	C12144	↑	Sphingolipid metabolism

↑ refers to the increase of the level of the metabolite in the model group compared to the control group, while ↓ refers to the decrease; ^a^ LysoPC(18:1) has two isomers: LysoPC(18:1(11Z)) and LysoPC(18:1(9Z)) and these cannot be distinguished based on the present data.

**Table 2:** Chemical information of compounds in V-kansui.

Symbol	Molecule Name	OB (%)	DL
GE01	citric acid	56.22	0.05
GE02	OXL	29.68	0.01
GE03	24-Methylenecycloartanol	10.4	0.79
GE04	*β*-sitosterol	5.84	0.71
GE05	20-*O*-(2,3-dimethylbutanoyl)-13-*O*-dodecanoylingenol	24.17	0.61
GE06	3-*O*-Benzoyl-20-deoxyingenol	12.27	0.8
GE07	3-*O*-benzoyl-13-*O*-dodecanoylingenol	28.74	0.57
GE08	5-*O*-Benzoyl-20-deoxyingenol	13.52	0.79
GE09	[(1*S*,2*R*,5*S*,6*R*)-6-methyl-2-methylol-norpinan-6-yl]methanol	24.87	0.07
GE10	Euphorbetin	35.89	0.54
GE11	(3*S*,5*R*,10*S*,13*R*,14*R*,17*R*)-17-[(1*R*)-1,5-dimethyl-4-methylenehexyl]-4,4,10,13,14-pentamethyl-2,3,5,6,7,11,12,15,16,17-decahydro-1*H*-cyclopenta[a]phenanthren-3-ol	42.37	0.77
GE12	Euponin	18.64	0.49
GE13	Karacolidine	60.53	0.71
GE14	20-*O*-Benzoyl-13-*O*-dodeeanoyl ingenol	28.65	0.56
GE15	(1*S*,4a*S*,10a*R*)-7-isopropyl-1,4a-dimethyl-5,8-dioxo-2,3,4,9,10,10a-hexahydrophenanthrene-1-carboxylic acid	29.08	0.35
GE16	kansuinin A	44.52	0.55
GE17	kansuiphorin A	21.67	0.22
GE18	kansuiphorin B	19.16	0.2
GE19	NSC 403164	8.51	0.75
GE20	Euphol	42.12	0.75
GE21	20-OD-ingenol Z	32.05	0.85
GE22	Kanziol	41.65	0.75
GE23	Glycerite	14.97	0.03
GE24	3-*O*-(2,3-Dimethylbutanoyl)-13-*O*-decanoyl ingenol	24.75	0.71
GE25	3-*O*-(2,3-Dimethylbutanoyl)-13-*O*-dodecanoyl-20-*O*-acetylingenol	25.44	0.54
GE26	3-*O*-(2,3-Dimethylbutanoyl)-13-*O*-dodecanoyl-20-deoxyingenol	30.82	0.65
GE27	3-*O*-(2,3-dimethyl-butanoyl)-13-dodecanoylingenol	24.3	0.63
GE28	Isoscopoletin	23.46	0.08
GE29	Scopoletol	27.77	0.08
GE30	palmitic acid	19.3	0.1
GE31	HMF	45.07	0.02

**Table 3:** 12 intersection targets with average shortest path length and betweenness centrality.

Genes	Description	Average Shortest Path Length	Betweenness Centrality	R
HSP90AA1	Heat shock protein HSP 90-alpha	2.17	0.06665	0.0000
PCNA	proliferating cell nuclear antigen	2.37	0.00782	0.1193
ANXA2	annexin A2	2.40	0.01316	0.1352
PRDX6	peroxiredoxin 6	2.53	0.02880	0.1455
PC	pyruvate carboxylase	2.61	0.00365	0.1696
ALB	albumin	2.61	0.01804	0.1797
SOD2	superoxide dismutase 2	2.63	0.02969	0.2294
APOA1	apolipoprotein A-I	2.68	0.01036	0.3243
FGA	fibrinogen alpha chain	2.80	0.00247	0.4764
CTH	cystathionase	3.07	0.00195	0.6409
AKR1C2	aldo-keto reductase family 1, member C2	3.15	0.00131	0.8362
CES1	carboxylesterase 1	3.64	0.00184	0.8523

## Supplementary Materials

The following are available online at http://www.mdpi.com/1420-3049/23/12/3246/s1, Figure S1: The representative base peak intensity chromatograms of serum samples (A–D) from four groups by UPLC-Q-TOF in positive (Left) and negative (Right) modes, Figure S2: Representative base peak intensity chromatograms of urinary samples (E-H) of four groups by UHPLC-Q-TOF in positive (Left) and negative (Right) modes, Figure S3: The response permutation test plots (*n* = 200) for the PLS-DA models for serum (A) and urine samples (B) in positive mode, Figure S4: The average relative intensity changes of urine endogenous metabolites from different groups, Table S1: UPLC-Q-TOF-MS data for Identification results of potential biomarkers, Figure S5: The network of potential metabolites for pathway-based genes, Figure S6: The extended protein-protein network of metabolites-related genes, Figure S7: The compound-target network representing the effect of treating malignant ascites by V-kansui, Figure S8: The compound-target-metabolite network.
