# Supplementary material for: Interpretation of Euphorbia Kansui Stir-Fried with Vinegar Treating Malignant Ascites by a UPLC-Q-TOF/MS Based Rat Serum and Urine Metabolomics Strategy Coupled with Network Pharmacology
Source: Molecules. 2018 Dec 7;23(12):3246. doi: 10.3390/molecules23123246 (PMC6322356; doi:10.3390/molecules23123246)
Supplement: Supplementary file 1 [file molecules-23-03246-s001.pdf]

molecules

Supplementary Materials

Interpretation of *Euphorbia kansui* stir-fried with vinegar treating malignant ascites by a UHPLC-Q-TOF based rat serum and urine metabolomics strategy coupled with network analysis

Yi Zhang [1], Jing Gao [1], Qiao Zhang [1], Wei-Feng Yao [1,*], Bei-Hua Bao [1], Li Zhang [1,*] and Yu-Ping Tang [2]

[1] Jiangsu Key Laboratory for High Technology Research of TCM Formulae, National and Local Collaborative Engineering Center of Chinese Medicinal Resources Industrialization and Formulae Innovative Medicine and Jiangsu Collaborative Innovation Center of Chinese Medicinal Resources Industrialization, Nanjing University of Chinese Medicine, Nanjing, 210023, China.

[2] College of Pharmacy and Shaanxi Collaborative Innovation Center of Chinese Medicinal Resources Industrialization, Shaanxi University of Chinese Medicine, Xi'an 712046, Shaanxi Province, China.

* Correspondence: yaowf@njucm.edu.cn (W. Y.); zhangli@njucm.edu.cn (L. Z.); Tel.: +86-258-581-1519 (W. Y. and L. Z.)

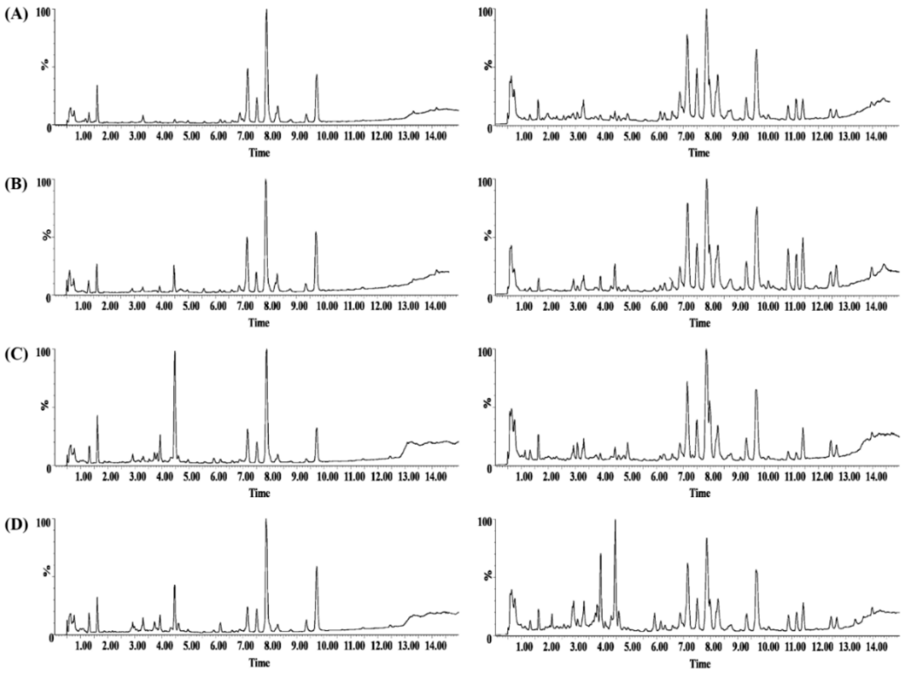

Figure S1. The representative base peak intensity chromatograms of serum samples (A-D) from four groups by UPLC-Q-TOF in positive (Left) and negative (Right) modes. A-D represent the Control, Model, Kansui, and V-kansui groups, respectively.

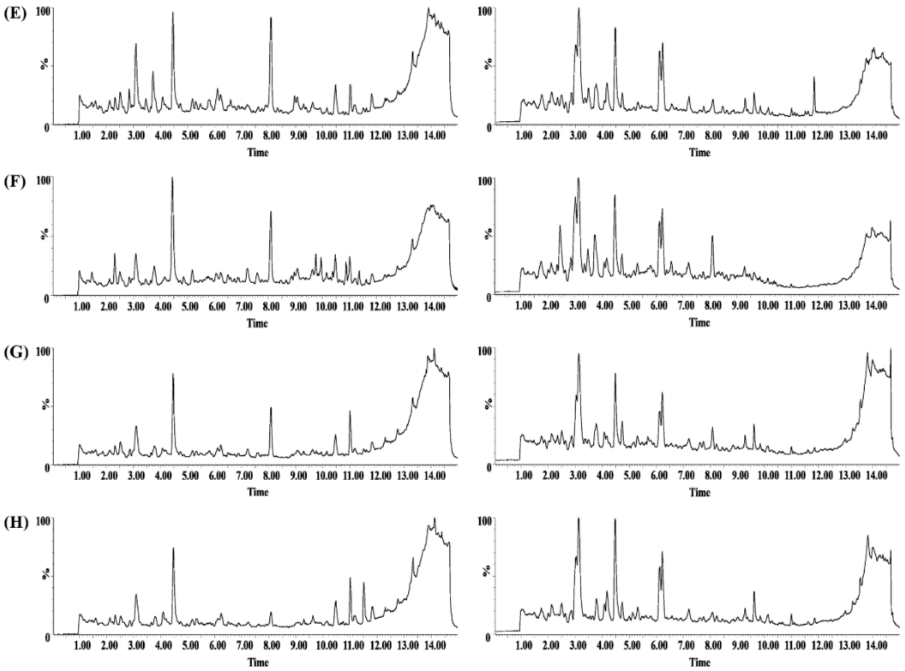

Figure S2. Representative base peak intensity chromatograms of urinary samples (E-H) of four groups by UHPLC-Q-TOF in positive (Left) and negative (Right) modes. E-H represent the Control, Model, Kansui, and V-kansui groups, respectively.

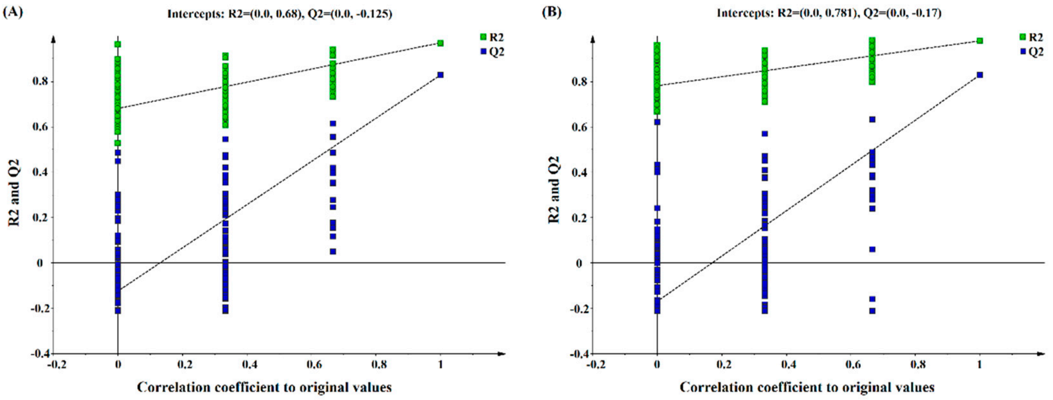

Figure S3. The response permutation test plots (n=200) for the OPLS-DA models for serum (A) and urine samples (B) in positive mode.

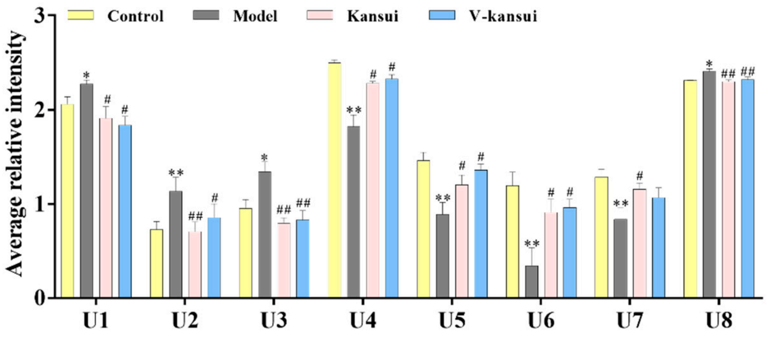

Figure S4. The average relative intensity changes of urine endogenous metabolites from different groups. Comparing the model group with the control group, * indicates $p < 0.05$, and ** indicates $p < 0.01$.; Comparing

the kansui group and V-kansui group with the model group, # indicates $p < 0.05$, and ## indicates $p < 0.01$indicates $p < 0.05$.

Table S1. UPLC-Q-TOF-MS data for Identification results of potential biomarkers

Metabolites	RT	Adduct	*m/z*	Mass error (ppm)	Fragments	Databases/Literatures
Indoleacetaldehyde	1.18	M+H	160.0698	8.7	145, 117	METLIN
Taurocholic acid	2.88	M-H	514.2942	5.0	124	METLIN
Taurochenodesoxycholic acid	3.91	M-H	498.2991	4.8	480, 462, 372, 124, 80	HMDB
Cholic acid	4.46	M-H	407.2886	2.4	343, 289	METLIN
Phytosphingosine	4.79	M+H	318.2912	-5.3	301, 300, 282, 197	METLIN, HMDB
Chenodeoxycholic acid	6.15	M-H	391.2934	2.0	373, 345, 268, 185	MASSBANK
LysoPC(18:1)	8.27	M+H	522.3438	-8.2	504, 283, 265, 241, 184, 166, 104	HMDB, METIN, [1,2]
Docosahexaenoic acid	10.88	M-H	327.2400	-0.6	283, 229	METLIN
5-Hydroxy-6-methoxyindole glucuronide/6-Hydroxy-5-methoxyindole glucuronide	3.73	M+H	340.0978	7.1	322, 164, 146, 132	HMDB, METLIN, [3]
Prostaglandin G_2	6.46	M+FA-H	413.2165	-9.0	287, 235, 113	METLIN, HMDB
10-Formyltetrahydrofolate	6.71	M-H	472.1692	7.0	455, 428, 293	HMDB
5-*L*-Glutamyl-taurine	7.22	M+H	255.0563	-3.5	237, 209, 194, 135, 126, 109	HMDB, METLIN, [4,5]
Riboflavin	8.96	M+H	377.1403	5.6	294, 243, 158	METLIN
Androstenedione	9.07	M+FA-H	331.1939	2.1	285, 267, 269	HMDB
11b-Hydroxyprogesterone	9.64	M-H	331.1973	-4.2	313, 287	HMDB
Phytosphingosine	10.48	M+H	318.2924	-1.6	300, 301, 282, 197	METLIN, HMDB

References:

[1] Shi, J.; Zhou, J.; Ma, H.; Guo, H.; Ni, Z.; Duan, J. A.; Qian, D. An in vitro metabolomics approach to identify hepatotoxicity biomarkers in human L02 liver cells treated with pekinenal, a natural compound. Analytical and bioanalytical chemistry 2016, 408, 1413-1424.

[2] Zhang, X.; Choi, F. F.; Zhou, Y.; Leung, F. P.; Tan, S.; Lin, S.; Bian, Z. Metabolite profiling of plasma and urine from rats with TNBS-induced acute colitis using UPLC-ESI-QTOF-MS-based metabonomics - a pilot study. The FEBS journal 2012, 279, 2322-2338.

[3] Zhao, T.; Zhang, H.; Zhao, T.; Zhang, X.; Lu, J.; Yin, T.; Li, P. Intrarenal metabolomics reveals the association of local organic toxins with the progression of diabetic kidney disease. Journal of pharmaceutical and biomedical analysis 2012, 60, 32-43.

[4] Chen, Y.; Duan, J. A.; Guo, J.; Shang, E.; Tang, Y.; Qian, Y.; Liu, P. Yuanhuapine-induced intestinal and hepatotoxicity were correlated with disturbance of amino acids, lipids, carbohydrate metabolism and gut microflora function: a rat urine metabonomic study. Journal of Chromatography B 2016, 1026, 183-192.

[5] Qi, Y.; Pi, Z.; Liu, S.; Song, F.; Lin, N.; Liu, Z. A metabonomic study of adjuvant-induced arthritis in rats using ultra-performance liquid chromatography coupled with quadrupole time-of-flight mass spectrometry. Molecular Biosystems 2014, 10, 2617-2625.

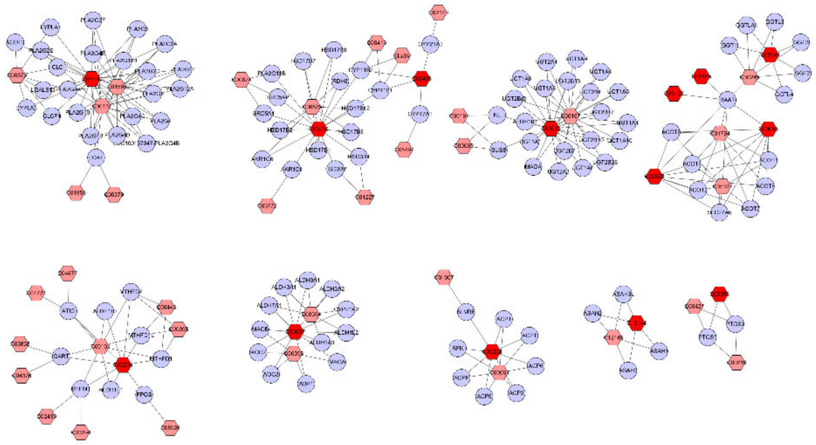

Figure S5. The network of potential metabolites for pathway-based genes. The red nodes represent the potential metabolites and grey nodes represent the related genes.

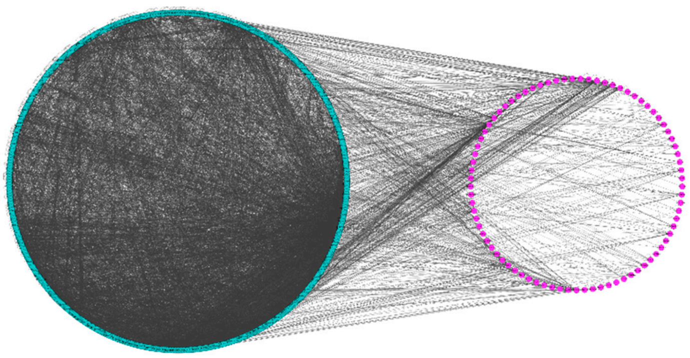

Figure S6. The extended protein-protein network of metabolites-related genes. The blue and pink nodes represent adjacent proteins and metabolite-related proteins, respectively.

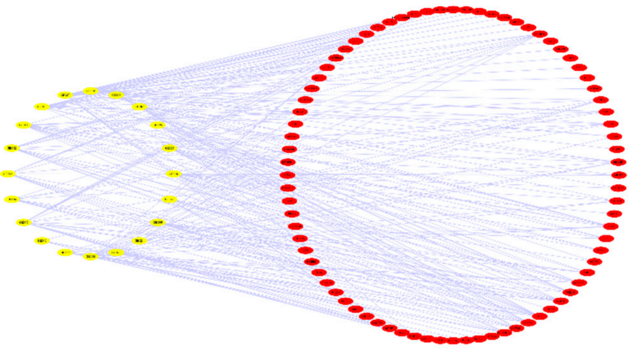

Figure S7. The compound-target network representing the effect of treating malignant ascites by V-kansui. The yellow and red nodes represent compounds and disease-related proteins, respectively.

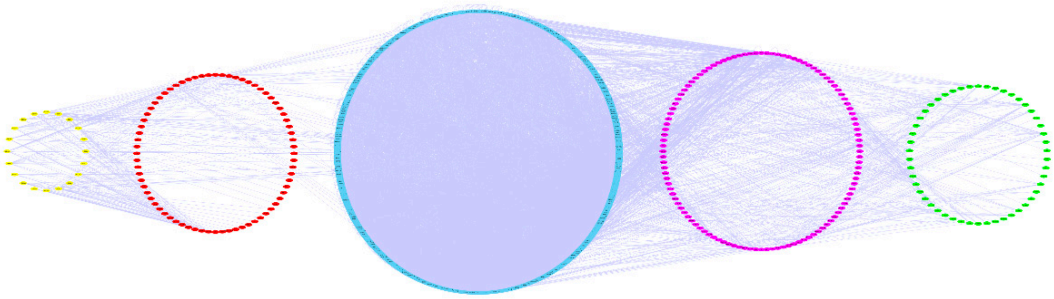

Figure S8. The compound-target-metabolite network. The yellow, red, blue, pink and green nodes represent the active compounds, targets, adjacent proteins, pathway genes and potential metabolites, respectively.

© 2018 by the authors. Licensee MDPI, Basel, Switzerland. This article is an open access article distributed under the terms and conditions of the Creative Commons Attribution (CC BY) license (http://creativecommons.org/licenses/by/4.0/).
